# Medical gases and hypertensive disorders of pregnancy: therapeutic mechanisms and research advances

**DOI:** 10.4103/mgr.MEDGASRES-D-26-00014

**Published:** 2026-09-12

**Authors:** Zhiguang Xu, Geng Tian, Di Zhou, Fuju Wu

**Affiliations:** 1 Department of Gynecology and Obstetrics, The Second Hospital of Jilin University, Jilin University, Changchun, Jilin Province, China; 2 Department of Ultrosound, The Second Hospital of Jilin University, Jilin University, Changchun, Jilin Province, China

**Keywords:** carbon monoxide, hydrogen sulfide, hyperbaric oxygen, hypertensive disorders of pregnancy, medical gas, nitric oxide, oxidative stress, placental perfusion, preeclampsia, preeclampsia pathophysiology

## Abstract

Hypertensive disorders during pregnancy pose a serious threat to the health of both mothers and infants, yet the range of available clinical treatments is limited. This review summarizes the mechanisms and recent advances in the use of medical gases, such as nitric oxide, hyperbaric oxygen, carbon monoxide and hydrogen sulfide, in the treatment of this condition. These gaseous molecules have unique protective effects, including regulating vascular tone, improving placental blood perfusion and inhibiting oxidative stress and inflammatory responses. However, their clinical application faces major challenges, including precise dose control, optimization of delivery methods and assessment of long-term safety. While this review suggests that medical gas therapy provides new avenues for research and potential applications in the management of hypertensive disorders of pregnancy, it also highlights the need for further basic and clinical studies to clarify their precise mechanisms.

## Introduction

Hypertensive disorders of pregnancy (HDP), comprising gestational hypertension and preeclampsia, are now recognized as significant contributors to maternal morbidity and mortality.^1^ With rising rates of obesity, chronic hypertension, and advanced maternal age, the burden of HDP is expected to increase.^2^

Medical gas is widely used in various fields of clinical and basic medicine,^3^ as illustrated in **Figure 1**. In recent years, medical gases such as nitric oxide (NO), hyperbaric oxygen (HBO), carbon monoxide (CO), and hydrogen sulfide (H_2_S) have gained prominence in research on the treatment of pregnancy-induced hypertension due to their unique physiological regulatory properties. Research indicates that these gas molecules exert protective effects through multiple mechanisms, including the regulation of vascular tone, the improvement of placental tissue perfusion, and the suppression of oxidative stress and inflammatory responses. Even though most contemporary research remains in the fundamental experimental or nascent clinical trial stages, further exploration holds promise for pioneering new treatment pathways for this condition. This review examined the research value and application potential of medical gas therapy in treating HDP. We also identified key scientific issues and technical challenges requiring immediate attention. Furthermore, we offered a theoretical basis and research directions for the subsequent development of personalized and precise treatment strategies, as well as the clinical translation of medical gas therapy in treating HDP.

**Figure 1:**
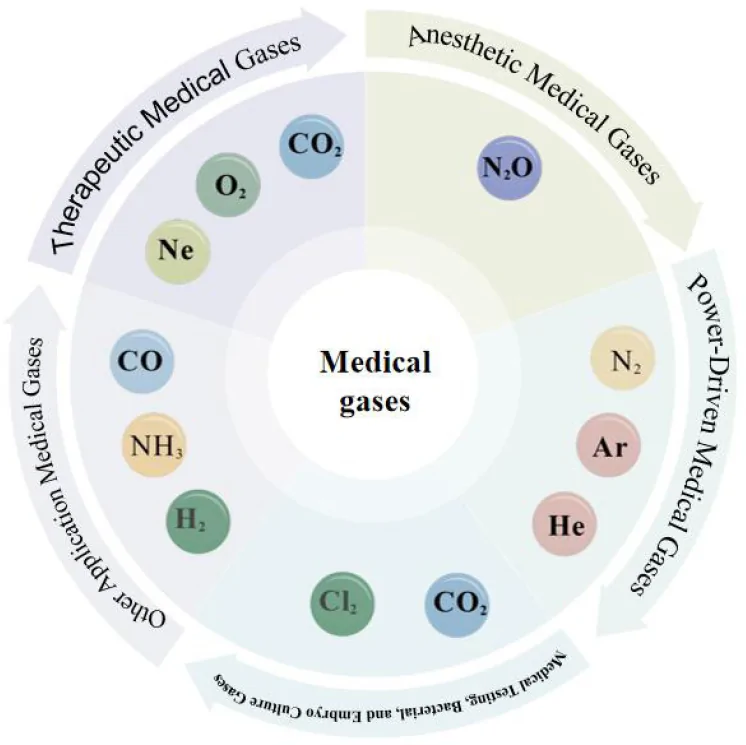
Classification of medical gases. Created with Microsoft PowerPoint. Ar: Argon; Cl_2_: chlorine; CO: carbon monoxide; CO_2_: carbon dioxide; CO_2:_ carbon dioxide; H_2_: hydrogen; He: helium; N_2_: nitrogen; N_2_O: nitrous oxide; Ne: neon; NH3: ammonia; O2: oxygen.

## Search Strategy

This review was conducted in accordance with the Preferred Reporting Items for Systematic Reviews and Meta-Analyses (PRISMA) guidelines (**Figure 2**).^4^ We conducted a systematic literature search in PubMed, X-Mol and ScienceDirect from database inception to March 2026, with no language restrictions initially (final inclusion limited to English articles as per eligibility criteria). The key search terms used were “hypertensive disorders of pregnancy,” “medical gases,” “nitric oxide,” “hyperbaric oxygen,” “carbon monoxide,” “Placental perfusion,” “Preeclampsia,” “Oxidative stress,” “hydrogen sulfide,” “mechanisms,” and “research advances.”

**Figure 2:**
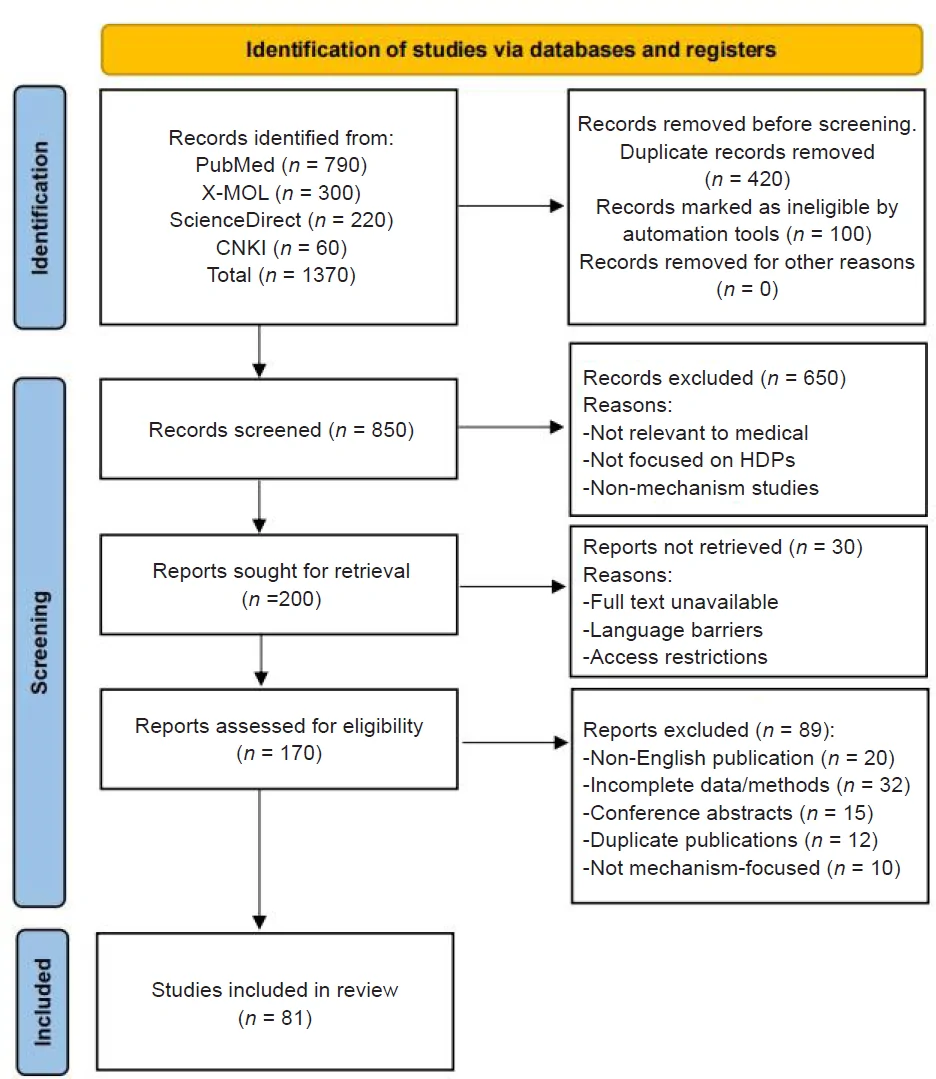
PRISMA 2020 flow diagram. HDP: Hypertensive disorders of pregnancy; PRISMA: Preferred Reporting Items for Systematic Reviews and Meta-Analyses.

All the studies related to the keywords were considered and subsequently screened according to the following eligibility criteria: (1) articles in English; and (2) articles on mechanism of medical gases on pregnancy-induced hypertension. The exclusion criteria included (1) reviews with insufficient methodological rigor or original studies with incomplete data; (2) conference papers; (4) duplicate studies; and (5) failure to provide detailed methodology or data.

## Pathogenesis of Hypertensive Disorders of Pregnancy

HDP arises from a complex interaction that involves the placenta, the immune system, the vascular system, and metabolism.^2^ Although a full understanding of the exact pathophysiological mechanisms behind HDP remains lacking, researchers have identified several processes as key initiators and exacerbators of this serious and complex condition.

### Impaired spiral artery remodeling

Insufficient trophoblast invasion and incomplete remodeling of the spiral arteries are the pathophysiological hallmarks of early-onset preeclampsia.^5^ The fundamental pathogenesis of preeclampsia mainly comes from the abnormal remodeling of spiral arteries inside the placenta, a process that begins in early pregnancy. During a normal pregnancy, extravillous trophoblasts effectively complete pseudoangiogenesis, differentiate into an endothelial-like phenotype, and deeply invade the spiral arteries located in the inner third of the uterine myometrium. By expressing critical endothelial adhesion markers like VE-cadherin and integrins α1β1 and αVβ3, these trophoblasts facilitate the physiological remodeling of spiral arteries.^6^ This remodeling process turns high-resistance, small-caliber muscular arteries into low-resistance, high-capacity vessels whose average diameter reaches 500 μm after remodeling. This structural change significantly enhances placental blood flow and stabilizes maternal blood flow velocity at roughly 10 cm/s, thereby ensuring proper maternal-fetal exchange.^7^ In cases of preeclampsia, however, the invasive differentiation of extravillous trophoblasts becomes severely impaired. These cells only infiltrate as far as the decidual layer, which prevents normal pseudoangiogenic transformation and leads to incomplete spiral artery remodeling with an average diameter of only about 200 μm, thereby causing proximal arterial lumen stenosis. This structural abnormality has been shown to produce severe uteroplacental perfusion insufficiency.

Placental ischemia and hypoxia activate complex molecular response mechanisms.^8^,^9^,^10^ One particularly important feature is the sustained expression of hypoxia-inducible factors (HIF-1α and HIF-2α), which are critical components in this process. During early normal pregnancy, the placenta exists in a physiologically hypoxic environment where increased HIF-1α expression promotes trophoblast cell proliferation and the expression of angiogenesis-related genes such as vascular endothelial growth factor and erythropoietin. By roughly gestational week 9, once placental-fetal circulation establishes and oxygenation improves, HIF-1α levels undergo physiological downregulation. In preeclamptic placentas, however, the regulatory processes involving HIF-1α and HIF-2α become impaired even when oxygenation increases.^11^ This persistently high expression state signals placental stress. Abnormal activation of the HIF signaling pathway directly drives excessive production of anti-angiogenic factors, especially soluble fms-like tyrosine kinase-1 (sFlt-1) and soluble endothelin.^12^,^13^ Elevated levels of these proteins appear in both *in vitro* and *in vivo* models of placental hypoxia. Other mechanisms, such as Semaphorin 3B, also impede trophoblast differentiation and invasive capacity by inhibiting the vascular endothelial growth factor signaling pathway. The active factors released from the ischemic placenta enter the maternal circulation, thereby triggering systemic vascular endothelial dysfunction, intense inflammatory responses, and oxidative stress. This process ultimately produces a series of maternal clinical manifestations that include hypertension, proteinuria, and glomerular endothelial hyperplasia; in certain cases, these may progress to HELLP (hemolysis, elevated liver enzymes, and low platelet count) syndrome. This observation aligns with the two-stage pathogenesis theory of preeclampsia, meaning the disease progresses from early placental perfusion abnormalities to late-stage maternal syndrome.^13^ Although the specific upstream mechanisms behind sustained HIF overexpression remain incompletely understood, the catechol-O-methyltransferase-catalyzed production of 2-methoxyestradiol may play a contributing role.

Abnormal placental development and abnormal remodeling of uterine spiral arteries are central to preeclampsia pathogenesis. The molecular mechanisms behind this phenomenon involve complex interactions across multiple levels, including trophoblast dysfunction, abnormal HIF signaling pathways, and a disrupted angiogenesis balance.

### Vascular endothelial dysfunction

The pathogenesis of systemic endothelial dysfunction in preeclampsia has been fully explained and falls into two distinct phases. The initial phase takes place during placental formation, where inadequate trophoblast invasion of the spiral arteries stops the placental bed arteries from switching from high-resistance to low-resistance vessels. This inadequacy then triggers local ischemia-reperfusion injury and oxidative stress. This localized injury subsequently activates the second phase, which features an imbalance in the production of pro-angiogenic and anti-angiogenic factors like placental growth factor (PLGF) and soluble sFlt-1. Such an imbalance leads to systemic inflammation, endothelial cell activation, systemic oxidative stress, and abnormal endothelial NO production. Clinical manifestations of preeclampsia appear when this vascular endothelial activation and dysfunction simultaneously affect organs, including the liver, kidneys, brain, and placenta.^14^,^15^,^16^,^17^ During the placental stress response, production of anti-angiogenic factors such as sFlt-1 and soluble endoglin rises, whereas pro-angiogenic factors like PLGF fall. This imbalance disrupts endothelial function, promotes vasoconstriction, and leads to maternal hypertension along with end-organ damage. The elevated sFlt-1/PLGF ratio has been studied as a possible biomarker for disease severity and progression.^18^ Preeclampsia is a pregnancy syndrome defined by hypertension and proteinuria that occurs after 20 weeks of gestation, affecting 3–5% of all pregnancies.^19^ Disease progression leads to maternal and fetal complications that include renal failure, cerebral edema, hepatic failure, low birth weight, preterm birth, and mortality.^19^ Although the pathophysiology of preeclampsia remains incompletely understood, evidence indicates that impaired trophoblast invasion and failure of uterine artery dilation cause high resistance inside the uteroplacental circulation. Reduced uteroplacental perfusion has been shown to produce placental hypoxia, which then induces factor release into the maternal circulation and triggers systemic endothelial dysfunction.

### Inflammation and immune activation

Inflammatory responses play a critical role in HDP.^20^,^21^,^22^ The maternal immune system usually undergoes adaptive modifications to tolerate the semi-allogeneic fetus, yet gestational hypertension disrupts this balance. During normal pregnancy, maternal immune tolerance to the semi-allogeneic fetus is maintained by regulatory T cells, anti-inflammatory macrophages, and a Th2-dominant profile. In gestational hypertension, this tolerance breaks down via a pro-inflammatory shift that impairs placental development, induces oxidative stress and anti-angiogenic factor release, and ultimately causes endothelial dysfunction and hypertension.^22^ Immune cell activation becomes heightened for cells, including dendritic cells, natural killer cells, helper T cells, and B cells, whereas the regulatory T cell population shrinks. This pro-inflammatory shift triggers cytokine release, leading to trophoblast dysfunction and placental hypoplasia. B cells specifically produce autoantibodies against the angiotensin II type 1 receptor, which stimulate vasoconstriction and further worsen inflammation and oxidative stress; these processes are integral to gestational hypertension pathophysiology.^23^ The resulting immune imbalance not only disrupts placental development but also contributes to systemic vascular dysfunction, thereby amplifying the characteristic inflammatory and hypertensive cascade seen in gestational hypertension.^24^,^25^,^26^,^27^

### Excessive oxidative stress

Gestational hypertension commonly links to placental ischemia, which then increases reactive oxygen species (ROS) production to a level that overwhelms antioxidant defense mechanisms.^20^,^21^,^22^,^23^,^28^ The accumulation of ROS is associated with lipid peroxidation, protein oxidation, and DNA damage, which in turn exacerbates endothelial dysfunction. The presence of oxidative stress in this environment has been demonstrated to facilitate the release of placental factors into the maternal circulation, thereby triggering systemic inflammation and vascular injury.^24^,^25^,^26^,^27^,^29^ Collectively, these oxidative stress-driven processes worsen both placental and systemic dysfunction, playing a pivotal role in the progression and severity of hypertensive disorders during pregnancy.

During early normal pregnancy, the placenta exists in a physiologically hypoxic environment where moderate ROS levels appear.^11^ Antioxidant defense systems such as superoxide dismutase, catalase, and glutathione peroxidase effectively maintain redox balance. Moderate oxidative stress may even contribute to normal cellular signaling and placental development. In preeclampsia cases, however, inadequate uterine spiral artery remodeling leads to persistent placental ischemia-reperfusion injury.^9^ This pathological process triggers explosive ROS production from several sources that include mitochondrial respiratory chain activation, nicotinamide adenine dinucleotide phosphate oxidase, xanthine oxidase, and endothelial NO synthase (NOS) dysfunction.^24^ ROS levels consequently far exceed the scavenging capacity of the antioxidant system, thereby producing a severe redox imbalance. This excessive oxidative stress creates a vicious cycle with inflammation. ROS promote pro-inflammatory cytokine release (e.g., tumor necrosis factor-α and interleukin-6) and activate monocytes and neutrophils. At the same time, anti-inflammatory mediator release (e.g., interleukin-10) and antioxidant capacity both diminish.^25^,^26^,^27^,^29^ Oxidative stress also promotes polyunsaturated fatty acid peroxidation, which generates large amounts of lipid peroxides and toxic aldehydes. These compounds then induce cellular dysfunction and apoptosis, resulting in an imbalanced thromboxane A2 to prostacyclin ratio that worsens vasoconstriction and platelet aggregation.^29^,^30^,^31^

Excessive oxidative stress directly damages syncytiotrophoblast cells, leading to inflammatory mediators, exosomes, anti-angiogenic factors (such as sFlt-1), and acellular fetal DNA release into the maternal circulation.^26^ These placental-derived factors trigger systemic vascular endothelial dysfunction and inflammatory responses, which ultimately manifest as clinical symptoms like hypertension and proteinuria.^8^,^26^ The heme oxygenase-1 (HO-1) protective mechanism further complicates this process. Preeclampsia features a marked reduction in placental HO-1 expression, which diminishes the vasodilatory and antioxidant effects of its catalytic products CO and bilirubin.^32^,^33^ Existing literature confirms that inducing HO-1 expression mitigates placental ischemia-induced hypertension. Conversely, *HO-1* gene knockout induces preeclampsia-like manifestations.^10^,^33^

Researchers have also hypothesized that endoplasmic reticulum stress and reduced activating transcription factor 3 expression may contribute to pathogenesis by promoting abnormal HIF and anti-angiogenic factor expression.^12^,^34^,^35^ Notably, in the early stages of preeclampsia, antioxidant defenses may be enhanced in a compensatory manner to protect the fetus, as exemplified by the upregulation of placental superoxide dismutase and catalase, which act to neutralize ROS and limit oxidative injury to the fetus.^25^,^36^ These defenses ultimately prove inadequate against ongoing oxidative stress.

### Mitochondrial dysfunction

Mitochondria inside trophoblast and endothelial cells play a crucial role in energy metabolism and redox balance. In HDP, mitochondrial dysfunction leads to impaired ATP production and excessive ROS generation. Previous studies have shown that alterations in mitochondrial respiration and bioenergetics within placental and endothelial cells serve as indicators of mitochondrial injury, and this injury significantly contributes to both placental and systemic endothelial dysfunction.^21^,^28^

### Epigenetic modifications

A mounting body of evidence suggests that epigenetic regulation, encompassing DNA methylation, histone modifications, and non-coding RNAs, plays a role in hypertensive disorders such as preeclampsia. As a representative example, dysregulated DNA methylation in key placental genes (e.g., FLT1, LEP) can alter their expression levels, leading to insufficient spiral artery remodeling, placental ischemia, and systemic endothelial injury, which are core pathological mechanisms underlying preeclampsia. These modifications have the potential to influence gene expression, which is involved in immune regulation, angiogenesis, and oxidative stress responses.^5^,^37^ Furthermore, the impact of the epigenetic landscape on fetal programming may lead to long-term risks of cardiovascular, renal, and metabolic diseases in offspring.^38^,^39^

The pathophysiology of HDP is inherently multifaceted and includes abnormal placental formation, dysregulated angiogenesis signaling, immune dysfunction, oxidative stress, and mitochondrial damage (**Figure 3**). These mechanisms interact synergistically and thereby create a toxic maternal environment, which then triggers additional cardiovascular and renal complications. Understanding these pathological pathways is crucial for developing innovative targeted therapies that aim to improve maternal and fetal outcomes.

**Figure 3:**
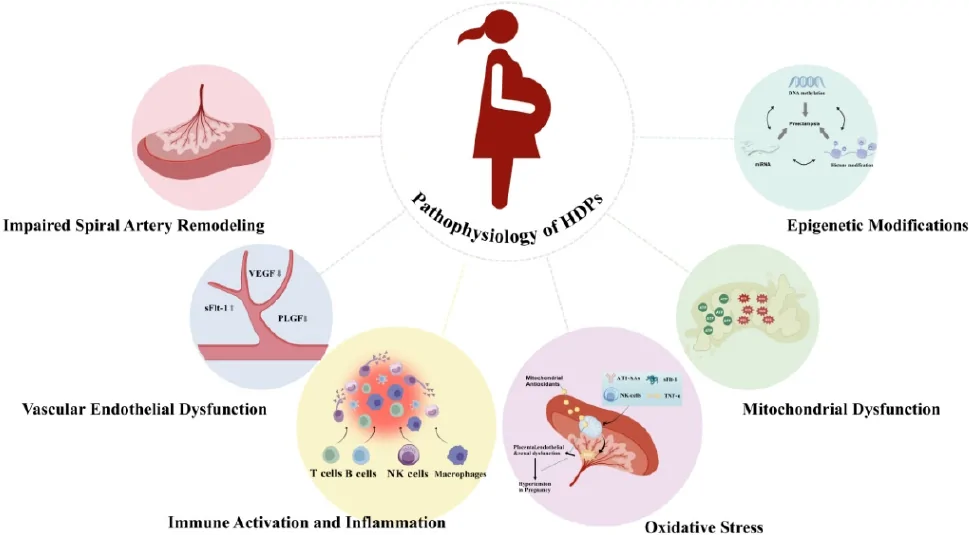
Pathogenesis of hypertensive disorders of pregnancy. Created with BioGDP. AT1-AA: Angiotensin II type 1 receptor autoantibody; HDP: hypertensive disorders of pregnancy; NK cells: natural killer cells; PLGF: placental growth factor; sFlt-1: soluble fms-like tyrosine kinase-1; TNF-α: tumor necrosis factor α; VEGF: vascular endothelial growth factor.

## Mechanism of Medical Gas Therapy in Hypertensive Disorders of Pregnancy

Given that the pathogenesis of hypertensive disorders of pregnancy is multifactorial, centrally involving impaired spiral artery remodeling, placental ischemia and hypoxia, excessive oxidative stress, inflammatory activation, endothelial dysfunction, and mitochondrial damage,^8^,^9^,^10^,^15^,^21^,^22^ it stands to reason that an ideal therapeutic strategy would need to simultaneously target these interconnected pathways. Intriguingly, medical gases including NO, HBO, CO and H_2_S exhibit pleiotropic effects that precisely correspond to these pathological hubs. Specifically, NO and H_2_S primarily counteract endothelial dysfunction and vasospasm through potent vasodilation and angiogenesis promotion.^40^,^41^,^42^ HBO directly alleviates placental hypoxia and reduces oxidative stress by enhancing oxygen delivery and upregulating antioxidant enzymes.^43^,^44^,^45^ CO, at strictly controlled low concentrations, exerts anti-inflammatory and cytoprotective effects via heme oxygenase-1 pathway activation,^32^,^33^ although higher concentrations may be detrimental.^46^,^47^,^48^ Thus, a mechanistic alignment exists between each gas and specific HDP pathologies, as summarized in **Table 1**.^32^,^33^,^40^,^42^,^43^,^44^,^45^,^49^,^50^,^51^,^52^ This targeted correspondence distinguishes medical gas therapy from conventional antihypertensive agents that lack such pleiotropic regulation of placental microenvironment.

**Table 1:** Correspondence between core pathological mechanisms of HDP and therapeutic targets of medical gases

Pathological hub of HDP	Corresponding medical gas	Primary therapeutic mechanism
Placental ischemia-hypoxia	HBO	Increases oxygen delivery, stabilizes endothelial barrier^43^,^44^
Endothelial dysfunction/vasospasm	NO	Potent vasodilation, restores NO bioavailability^40^,^49^
Oxidative stress	HBO, low-dose CO	Upregulates antioxidant enzymes, reduces ROS^32^,^33^,^45^
Inflammation	CO (low concentration), H_2_S	Anti-inflammatory cytokines, inhibits NF-κB^32^,^50^,^51^
Impaired angiogenesis	NO, H_2_S	Promotes VEGF, PLGF, and endothelial progenitor function^40^,^42^,^52^

CO: Carbon monoxide; H_2_S: hydrogen sulfide; HBO: hyperbaric oxygen; HDP: hypertensive disorders of pregnancy; NF-κB: nuclear factor κB; NO: nitric oxide; PLGF: placental growth factor; ROS: reactive oxygen species; VEGF: vascular endothelial growth factor.

### Nitric oxide

NO is a tiny gaseous signaling molecule that is produced through the L-arginine NOS pathway or through alternative enzymatic and non-enzymatic nitrate–nitrite–NO pathway.^53^,^54^,^55^ This free radical participates in vasodilation, neurotransmission, cellular signaling, immune responses, and many other biological processes. Among the three known NOS subtypes, inducible NOS (iNOS) is unique because it can produce large quantities of NO as part of the immune response to inflammatory stimuli like infection or immune activation, as well as oxidative stress. NO exerts a cytostatic effect on bacteria, fungi, and viruses.^56^,^57^ Furthermore, NO not only interacts with protein kinase A and C, the p42/p44 mitogen-activated protein kinase/extracellular signal-regulated kinase pathway, and other specific kinases and phosphatases via multiple mechanisms to modulate iNOS activity, but also directly inhibits iNOS expression and enzymatic function. This feedback ensures tight control of NO production to prevent excessive oxidative stress while maintaining physiological vasodilatory function.^58^

A healthy pregnancy comes with physiological vascular adaptations that include an increase in blood volume regulated by systemic vasodilation.^40^ NO plays a crucial role in this process by reducing peripheral vascular resistance. Under normal conditions, NO serves as the primary vasodilator in the placenta, where it facilitates placental vasodilation and regulates fetalplacental blood flow. NO also participates in systemic vasodilation and renal adaptation in the mother during pregnancy, so the physiological reduction in blood pressure largely depends on its vasodilatory effects.^49^,^59^ After embryo implantation, endothelial NOS and iNOS mediate the effects of NO on tissue remodeling, immune suppression, and vascular regulation within the fetalplacental interface.^60^,^61^ NO acts as a crucial mediator of angiogenesis, and it facilitates vascular development while playing an essential role in placental vasculature maturation during pregnancy. In preeclampsia and other HDP, systemic endothelial dysfunction leads to rapid NO scavenging by ROS, and this scavenging greatly reduces NO bioavailability. This reduced NO bioavailability is a key factor that contributes to the development and progression of preeclampsia and other hypertensive disorders of pregnancy. When placental vascular abnormalities occur, a NO deficiency can produce fetalplacental endothelial dysfunction, disrupted hemodynamic adaptation, and an imbalance in coagulation and fibrinolysis. In intrauterine growth restriction cases, this deficiency associates with inadequate trophoblast invasion of spiral arteries and subsequent placental vascular dysfunction.^62^ The pathogenesis of HDP shares similarities with intrauterine growth restriction, including impaired trophoblast invasion of spiral arteries during early placental development.^63^,^64^ When the arteries in the placental bed cannot transition from high-resistance to low-resistance vessels, local ischemia-reperfusion injury and oxidative stress result. This local injury then activates a secondary phase that disrupts angiogenesis and antiangiogenic factor production. Consequently, systemic inflammation, endothelial activation, systemic oxidative stress, and altered endothelial NO production follow. Clinical manifestations of HDP appear once this endothelial activation and dysfunction affect organs such as the liver, kidneys, brain, and placenta.

The placenta plays a crucial role in fetal programming by directly regulating blood flow, transporter activity, and fetal growth,^65^ whereas epigenetic mechanisms influence placental maturation and development.^66^ NO is recognized as a significant player in epigenetic fetal programming.^67^,^68^ NO is also central to angiogenesis during pregnancy because it acts as a downstream effector molecule for several proangiogenic factors, including vascular endothelial growth factor, fibroblast growth factor, and angiopoietin-1. NO may also negatively regulate the expression of these factors through the HIF-1 pathway.^69^

Compared to healthy nonpregnant women, women with uncomplicated pregnancies show elevated circulating levels of NO metabolites, specifically nitrite and nitrate.^70^ The observed reduction in NO markers during preeclampsia and gestational hypertension indicates that decreased NO bioavailability links to hypertensive disorders of HDP (**Figure 4**).

**Figure 4:**
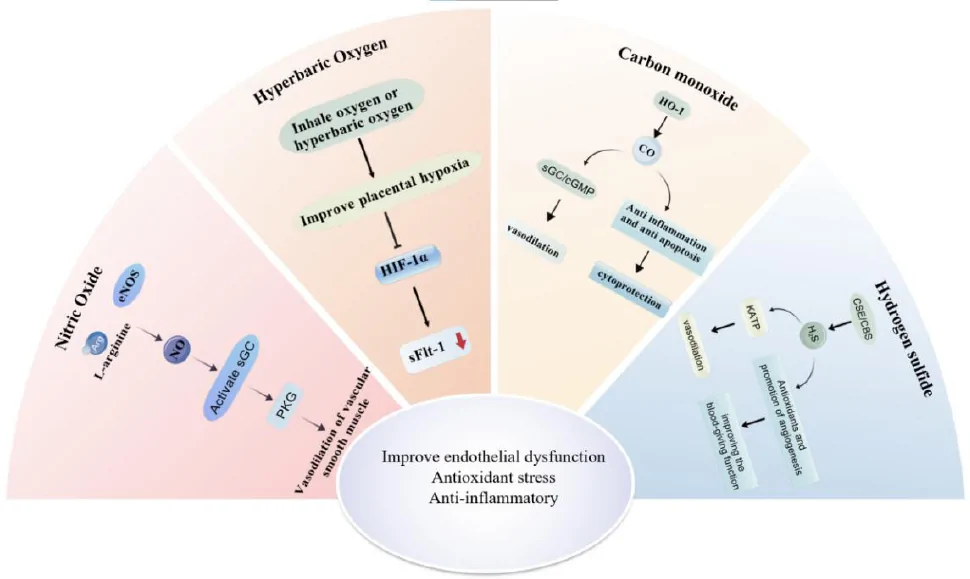
Mechanism of medical gas therapy in pregnancy-induced hypertension. Created with Microsoft PowerPoint. Arg: Arginine; CBS: cystathionine β-synthase; cGMP: cyclic guanosine monophosphate; CO: carbon monoxide; CSE: cystathionine γ-lyase; eNOS: endothelial nitric oxide synthase; H_2_S: hydrogen sulfide; HIF-1α: hypoxia-inducible factor 1α; HO-1: heme oxygenase-1; NO: nitric oxide; PKG: protein kinase G; sFlt-1: soluble fms-like tyrosine kinase-1; sGC: soluble guanylyl cyclase.

### Hyperbaric oxygen

HBO therapy significantly improves tissue hypoxia because it increases blood oxygen partial pressure and oxygen diffusion capacity.^43^ Oxygen therapy also inhibits fluid leakage into the interstitial space by stabilizing endothelial cells and reducing vascular permeability, thereby preventing inflammatory mediator extravasation.^71^ Research indicates that HBO can improve the pathological state of pregnancy-induced hypertension through multiple mechanisms. HBO significantly enhances oxygen supply to placental tissue, so it alleviates placental ischemia and hypoxia caused by incomplete spiral artery remodeling, thereby reducing oxidative stress and inflammatory responses.^44^ HBO upregulates antioxidant enzyme expression, such as superoxide dismutase and glutathione peroxidase, thus reducing ROS levels and mitigating lipid peroxidation together with endothelial injury.^72^ HBO also produces anti-inflammatory effects by reducing pro-inflammatory factor release (e.g., tumor necrosis factor-α and interleukin-6) while promoting anti-inflammatory cytokine production, thereby improving systemic inflammatory conditions.^51^ In experimental models using pregnant rats with hypertension, HBO intervention elicits a substantial blood pressure reduction and also notably increases placental and fetal weights.^73^ These observations suggest a potential improvement in intrauterine growth restriction.

A preliminary clinical study suggests that HBO therapy may enhance NO bioavailability, improve endothelial function,^52^ and promote normal placental vascular development through the regulation of angiogenic factor expression, such as vascular endothelial growth factor and PLGF. Although these findings are promising, HBO therapy remains in the preliminary research phase for treating pregnancy-induced hypertension. Further investigation must clarify the safety profile, therapeutic time window, and dose–response relationship of HBO therapy. Special caution is necessary regarding the potential risks of oxygen toxicity and free radical damage that come with high-concentration oxygen exposure (**Figure 4**).

### Carbon monoxide

In mothers with multiple pregnancies, elevated carboxyhemoglobin (COHb) levels during early pregnancy represent a risk factor for subsequent preeclampsia development.^74^ Research indicates that exposure to high CO concentrations during pregnancy associates with a slight increase in both mean systolic and diastolic blood pressure.^75^ This association mainly comes from documented vascular function impairment and/or atherosclerosis promotion. CO’s role in blood pressure regulation is multifaceted. In endothelial cells, CO inhibits NO formation, thereby blocking NO activity and reducing soluble guanylate cyclase responsiveness to NO, and this effect contributes to blood pressure elevation.^46^,^76^,^77^ Impaired NO formation or function in vascular smooth muscle cells raises blood pressure by reducing smooth muscle relaxation. Exogenous CO exposure also promotes endothelium-dependent vasoconstriction in isolated rat arterioles.^78^,^79^,^80^ Research conducted on indoor barbecue workers has indicated a positive correlation between COHb concentrations and carotid–intima–media thickness in individuals with high CO exposure,^47^ and this vascular remodeling mechanism is also applicable to the maternal vascular system in pregnancy, abnormal vascular thickening is a typical pathological feature of HDP, which directly leads to increased maternal peripheral vascular resistance and elevated blood pressure. Altered vascular function induced by CO exposure is closely associated with changes in HO synthesis, and this correlation is particularly evident in metabolic disorders associated with hypertension and ischemia/reperfusion injury,^46^ pathological states that are highly overlapping with the maternal systemic metabolic and vascular abnormalities in HDP. For instance, two experimental studies in rats with metabolic syndrome demonstrated that CO-induced endothelial dysfunction following high-salt diet treatment is HO-mediated,^48^,^81^ such endothelial dysfunction is a core pathophysiological link of HDP, and its occurrence in the maternal and placental vascular system will directly aggravate placental perfusion insufficiency and maternal hypertension. Consequently, in pregnancy, CO-induced alterations in vascular thickness, HO activity, and NO function may further compromise the relaxation of maternal and placental vascular endothelium, potentially resulting in elevated maternal blood pressure and exacerbating the pathological state of HDP. Furthermore, the inhalation of even low levels of CO results in tissue hypoxia in the maternal-fetal interface, as CO binds to hemoglobin with an affinity that is 250 times greater than that of oxygen,^82^ which shifts the oxygen-hemoglobin dissociation curve to the left and further reduces placental oxygen delivery, an effect that will aggravate placental ischemia and hypoxia, the primary initiating factor of HDP. Elevated maternal COHb concentrations also increase cardiac output by raising heart rate and/or cardiac contractility,^83^ a cardiovascular compensatory response to impaired oxygen-carrying capacity. For instance, an increase in the concentration of COHb from 0% to 15% has been shown to increase stroke volume,^84^ and an increase in COHb from 0% to 11% has been demonstrated to enhance cardiac contractility in rats.^85^ In addition, studies conducted on rats have indicated a significant increase in mean cardiac output at COHb levels ranging from 38% to 42%.^86^ An increase in maternal total blood volume may further serve as a compensatory mechanism that counteracts the reduced oxygen-carrying capacity of hemoglobin in the presence of CO,^87^ and the combined augmentation in cardiac output and blood volume will ultimately lead to molecular modification and vascular thickening of the maternal systemic circulation. This provides a partial explanation for the elevated blood pressure observed in pregnant women with excessive CO exposure (**Figure 4**).

### Hydrogen sulfide

H_2_S is a crucial endogenous signaling molecule that has multifaceted regulatory functions. This molecule localizes primarily in the cytoplasm and mitochondria, and mammalian tissues synthesize it endogenously via both enzymatic and non-enzymatic pathways. The primary enzymatic pathway involves three key enzymes. Cystathionine γ-lyase, the enzyme responsible for H_2_S production, is predominantly expressed in the cardiovascular system, liver, and kidneys, and it uses L-cysteine as its substrate to produce H_2_S. Cystathionine β-synthase shows high expression levels in the central nervous system (especially in astrocytes), and this catalyst facilitates the conversion of L-cysteine and L-homocysteine into H_2_S. 3-Mercaptopropionate S-transferase has been detected in both mitochondria and cytoplasm, where it functions together with cysteine aminotransferase to produce H_2_S. The reduction of thiosulfate and the conversion of other sulfur-containing substances also contribute to H_2_S production under certain physiological or pathological conditions. Many studies have demonstrated H_2_S therapeutic efficacy in various animal disease models, including hypertension, ischemic stroke, diabetes, fatty liver disease, acute kidney injury, and chronic renal fibrosis.^41^,^88^,^89^,^90^,^91^

H_2_S is synthesized in the placental and uterine vasculature, and there it plays a crucial role in vasodilation, angiogenesis, and placental function.^42^,^92^ In early-onset preeclampsia, cystathionine β-synthase expression (the enzyme responsible for H_2_S synthesis) drops significantly, which leads to decreased H_2_S production.^92^ Yadav et al.^50^ demonstrated that serum H_2_S levels are markedly decreased in sFlt-1-induced preeclampsia, and this decrease correlates with elevated mean arterial pressure together with reduced uterine blood flow. Treatment with the sustained-release H_2_S donor GYY4137 produces a significant mean arterial pressure reduction, a uterine blood flow increase, and a general alleviation of preeclampsia symptoms.^93^,^94^ These findings underscore the potential of GYY4137 as a therapeutic strategy for HDP (**Figure 4**).

In summary, the four medical gases exert protective effects against HDP through distinct yet interconnected mechanisms (**Table 2**). NO and H_2_S promote vasodilation and angiogenesis^40^,^41^,^42^,^50^; HBO alleviates placental hypoxia and oxidative stress^43^,^44^,^45^; low-dose CO provides anti-inflammatory and cytoprotective effects,^32^,^33^ whereas higher CO concentrations are detrimental.^46^,^47^,^48^ These complementary mechanisms support combination therapy because HBO may preserve NO bioavailability by reducing ROS,^45^ low-dose CO may complement vasodilation through its anti-inflammatory action,^32^,^51^ and NO together with H_2_S may synergize in promoting angiogenesis.^40^,^42^

**Table 2:** Comparative summary of therapeutic targets and core effects of four medical gases in HDP

Medical gas	Primary target site	Core therapeutic effect	Key mechanism	Dose-dependent consideration
Nitric oxide	Vascular endothelium, smooth muscle	Vasodilation, angiogenesis	sGC activation, VEGF/PLGF upregulation^40^,^49^	Generally beneficial; bioavailability reduced by ROS
Hyperbaric oxygen	Placental tissue, mitochondria	Alleviate hypoxia, reduce oxidative stress	Enhance O_2_ delivery, upregulate antioxidant enzymes^43^,^44^,^45^	Anti-oxidant *vs.* pro-oxidant
Carbon monoxide	Immune cells, HO-1 pathway	Anti-inflammatory, cytoprotective (low dose)	HO-1 activation, NF-κB inhibition^32^,^33^	protective (low) *vs.* vasoconstrictive/hypertensive (high)^46^,^47^,^48^
Hydrogen sulfide	Vascular smooth muscle, placenta	Vasodilation, angiogenesis, anti-inflammation	sGC or downstream signaling, VEGF promotion^41^,^42^,^50^	Generally beneficial; sustained-release donors preferred

HDP: Hypertensive disorders of pregnancy; HO-1: heme oxygenase-1; NF-κB: nuclear factor κB; NO: nitric oxide; O_2_: oxygen; PLGF: placental growth factor; ROS: reactive oxygen species; sGC: soluble guanylyl cyclase; VEGF: vascular endothelial growth factor.

## Limitations and Future Prospects

From a mechanistic perspective, various gases influence the pathophysiological processes of HDP through multiple pathways. Notably, NO and H_2_S primarily exert their effects by promoting vasodilation, enhancing placental blood flow, and inhibiting oxidative stress.^40^,^41^,^50^ In contrast, HBO focuses on alleviating tissue hypoxia and regulating redox balance.^44^,^45^ Furthermore, CO exhibits more complex effects; at low concentrations,^7^,^33^ it may exert positive impacts through anti-inflammatory and cytoprotective mechanisms, while high-concentration exposure can induce vasoconstriction and elevated blood pressure.^46^,^47^,^48^ This duality highlights the necessity for future studies to meticulously control gas concentration, timing of administration, and delivery methods to mitigate potential toxicity and maximize therapeutic benefits. They focus on ameliorating abnormal placental perfusion and alleviating placental ischemia-hypoxia status, which is a primary factor in the pathogenesis of HDP.^5^,^8^ Moreover, these gases regulate vascular function in two main ways: they either induce vasodilation directly or repair vascular endothelial damage.^40^,^41^,^50^ Through these mechanisms, the gases not only regulate peripheral vascular resistance to produce hypotensive effects, but they also exhibit antioxidant and anti-inflammatory properties.^44^,^50^,^51^ These effects are mediated through the scavenging of ROS, upregulation of antioxidant enzyme expression, and inhibition of pro-inflammatory factor release, thereby balancing inflammatory responses.^25^,^45^,^50^ Additionally, they modulate the microenvironmental homeostasis of the maternal-fetal interface by enhancing trophoblast cell function and promoting angiogenesis, ultimately supporting normal fetal development.^40^,^41^,^42^

Although the therapeutic potential of these four medical gases has been preliminarily validated, existing studies show numerous limitations that hinder clinical application. Most research remains confined to cellular experiments or animal models, so large-sample, multicenter randomized controlled trials are lacking. Clinical data on HBO treatment for HDP mostly consist of small-sample observations,^95^ and the clinical application of CO and H_2_S remains in the early exploratory phase with insufficient evidence-based medicine to support their efficacy and safety.^50^ A significant barrier to widespread clinical application is the high equipment cost. HBO treatment relies on HBO chambers whose price can exceed 2 million yuan, making such treatment difficult to implement in primary healthcare institutions.^95^,^96^ Developing targeted delivery systems for CO and H_2_S (e.g., placenta-specific donors) is both costly and technically challenging, which further limits their accessibility.^50^ Substantial individual variations in HDP patients regarding disease subtypes, gestational age at onset, and complications present additional challenges, so achieving consistent treatment outcomes becomes complicated.^18^,^69^ The interaction between signaling molecules and their shared placental mechanisms of action also requires further investigation.

Future research directions should focus on developing personalized treatment regimens, conducting large-scale multicenter clinical validations, exploring synergistic effects among different gases, and fostering technological innovations through device optimization. Precision intervention strategies should be tailored to patients’ biomarker profiles (e.g., sFlt-1/PLGF ratio, HIF-1α expression levels), disease subtypes, and gestational age. For severe placental ischemia, for example, HBO combined with low-dose H_2_S donors should be used, whereas NO precursors (e.g., L-arginine) should be prioritized for mild pregnancy-induced hypertension. Randomized controlled trials must systematically evaluate both short-term efficacy (including blood pressure control, proteinuria improvement, and placental blood flow recovery) and long-term safety (encompassing fetal growth and development as well as maternal postpartum cardiovascular disease risk) for all four gases. The synergistic potential of different gases warrants exploration, especially the complementary effects of NO and H_2_S in vasodilation and angiogenesis and the combined action of HBO and CO against oxidative stress. This approach aims to reduce individual gas dosages through combination therapy, thereby minimizing adverse reactions.

Investigating gas therapy application for early HDP intervention and prevention, together with multi-omics technologies to uncover epigenetic regulatory mechanisms, represents a significant research avenue. The interaction between gaseous signaling molecules and their synergistic mechanisms inside the placenta requires further examination. Both NO and H_2_S have demonstrated either synergistic or antagonistic effects in regulating vascular tone, for instance. Exploring their combined application could enhance therapeutic efficacy while reducing the adverse reactions that come with high-dose monotherapy.

No study to date has defined pregnancy-specific therapeutic windows or toxicity thresholds for NO, HBO, CO, or H_2_S in the HDP context despite a systematic literature search. This absence of fundamental safety and dosing data is a major barrier to clinical translation, and future dose-escalation studies in appropriate animal models must address this gap before clinical trials can responsibly begin.

Medical gas therapy has gained considerable research interest and applicability. Current studies face several challenges, including insufficient evidence, dosage regulation difficulties, and incomplete safety evaluations. Future research should focus on addressing these existing bottlenecks by developing personalized protocols, conducting large-sample clinical validations, fostering technical innovations, and elucidating underlying mechanisms. Such efforts will facilitate the transition of medical gas therapy from laboratory settings to clinical practice. Collaboration among different disciplines is crucial because it enhances the issue’s significance by involving medical specialists, healthcare providers, obstetricians, and engineers (particularly pharmacologists). Gases are expected to become a vital component of the HDP treatment system.

## Conclusion

HDP is a major health risk to mothers and fetuses, yet clinical treatment options are limited. Utilization of medical gas therapy for the prevention and management of HDP is a new possibility because of its unique vasoregulatory, anti-inflammatory, antioxidant, and cytoprotective effects. This review indicates that NO, HBO and H2S could enhance placental perfusion, alleviate endothelial dysfunction and inhibit oxidative stress, while CO may have protective effects at strictly low controlled concentrations. However, a critical takeaway from this review is that all four medical gases exhibit dose-dependent biphasic effects. The existing evidence contains unresolved contradictions, particularly regarding CO (cytoprotection *vs*. vasoconstriction/hypertension) and HBO (anti-oxidant *vs*. pro-oxidant effects). These dual roles underscore that generalized statements about therapeutic efficacy are insufficient without precise specification of dose, timing, and delivery method. Therefore, clinical application of these gases faces major challenges such as dose control, delivery optimization, and long-term safety evaluation. In summary, medical gas therapy for HDP offers significant research opportunities but remains at an early stage of clinical translation. Additional fundamental and clinical investigations are urgently needed to define the precise therapeutic windows that maximize benefit while avoiding toxicity, resolve current contradictions through systematic dose-response studies, and evaluate long-term maternal and fetal safety. Combining innovative strategies through interdisciplinary collaboration and translational medical research, gas therapy could play a significant role in the future as one of the components of comprehensive HDP management strategies to reduce maternal and perinatal mortality as well as complication rates.

**Facts**

1. Hypertensive disorders of pregnancy (HDP) is primarily driven by impaired spiral artery remodeling, leading to placental hypoxia and excessive oxidative stress.
2. Medical gases, particularly nitric oxide (NO) and hyperbaric oxygen (HBO), have consistently shown efficacy in reversing this ischemic microenvironment and mitigating reactive oxygen species production.
3. Beyond simple vasodilation, medical gases exert protective effects through pleiotropic pathways.
4. While NO and HBO are advancing, carbon monoxide (CO) and hydrogen sulfide (H_2_S) remain in the preclinical stage due to challenges in targeted delivery and dose control.

**Open questions**

1. What is the precise dose-response relationship and optimal treatment window for NO and HBO?
2. How can we overcome the systemic toxicity of CO and H_2_S to achieve placenta-specific delivery?
3. Is combined gas therapy (e.g., NO and HBO) superior to monotherapy in reducing severe complications such as eclampsia?
4. What are the potential drug–drug interactions when combining gaseous molecules with conventional antihypertensives?
5. What are the long-term cardiovascular and neurodevelopmental impacts of *in utero* exposure to medical gas therapy?

## Data Availability

*Not applicable.*
